# Molecular differentiation of commercial varieties and feral populations of oilseed rape (*Brassica napus *L.)

**DOI:** 10.1186/1471-2148-10-63

**Published:** 2010-03-01

**Authors:** Kathrin Pascher, Susanne Macalka, Domenico Rau, Günter Gollmann, Helmut Reiner, Josef Glössl, Georg Grabherr

**Affiliations:** 1 University of Vienna, Department of Conservation Biology, Vegetation Ecology and Landscape Ecology, Rennweg 14, A-1030 Vienna, Austria; 2 University of Natural Resources and Applied Life Sciences, Department of Applied Genetics and Cell Biology, Muthgasse 18, A-1190 Vienna, Austria; 3 Università degli Studi di Sassari, Dipartimento di Scienze Agronomiche e Genetica Vegetale Agraria, Via E De Nicola n 1, I-07100 Sassari, Italy; 4 University of Vienna, Department of Evolutionary Biology and Department of Limnology, Althanstraße 14, A-1090 Vienna, Austria; 5 Grünentorgasse 19/12, A-1090 Vienna, Austria

## Abstract

**Background:**

For assessing the risk of escape of transgenes from cultivation, the persistence of feral populations of crop plants is an important aspect. Feral populations of oilseed rape, *Brassica napus*, are well known, but only scarce information is available on their population dynamics, particularly in Central Europe. To investigate genetic diversity, origin and persistence of feral oilseed rape in Austria, we compared variation at nine polymorphic microsatellite loci in eight feral populations with 19 commercial varieties.

**Results:**

Overall, commercial varieties and feral populations showed a similar pattern of genetic variation and a similar level of observed heterozygosity. The two groups, however, shared less than 50% of the alleles and no multilocus genotype. A significant among-group (commercial varieties *versus *feral populations) component of genetic variation was observed (AMOVA: *F*_CT _= 0.132). Pairwise comparisons between varieties and feral populations showed moderate to very high genetic differentiation (*F*_ST _= 0.209 - 0.900). The software STRUCTURE also demonstrated a clear separation between commercial varieties and feral samples: out of 17 identified genetic clusters, only one comprised plants from both a commercial variety and feral sites.

**Conclusions:**

The results suggest that feral oilseed rape is able to maintain persistent populations. The feral populations may have derived from older cultivars that were not included in our analyses or perhaps have already hybridised with related crops or wild relatives. Feral populations therefore have to be considered in ecological risk assessment and future coexistence measures as a potential hybridisation partner of transgenic oilseed rape.

## Background

In the debate about the impact of transgenic plants on the environment, the containment of transgenes is a central issue [1]. Escape of transgenes could induce the evolution of new weeds or endanger the integrity of genetic resources in wild populations [2,3]. In gene transfer from crops to wild relatives, naturalized and feral populations of the crop plants themselves can form stepping stones for introgressive hybridization.

Oilseed rape, *Brassica napus *L., is an allotetraploid crop originating from hybridization between *Brassica oleracea *L. and *Brassica rapa *L. So far, there is no evidence that *B. napus *does exist as a wild plant but it frequently appears as a feral crop outside cultivation. It shows many traits typical for wild plants, such as secondary seed dormancy, adaptation of its seed germination to the annual cycle and the easy dehiscence of its fruits. Although oilseed rape is autogamous to a high degree, cross fertilization is common (12 to 55%) [4,5]. Carried by both vectors, wind and insects, *Brassica *pollen is able to pollinate flowers at distances of more than one kilometer [6,7]. A major concern for ecological risk assessment of transgenic oilseed rape is its potential for hybridization with related species [8,9]. Many species closely related to oilseed rape (e.g. *B. rapa, Diplotaxis *spp., *Hirschfeldia incana, Rhaphanus raphanistrum, Sinapis arvensis*) are found in agrotopes (field edges and meadows, loess slopes, shelterbelts, etc.) as well as ruderal habitats (road verges, railway embankments, slag heaps, etc.) [10]. Likewise, feral oilseed rape is a species of pioneer habitats, such as waste sites, cultivated grounds, rubble tips, arable fields, riverbanks, roadsides and tracks [11].

The persistence of feral populations therefore is an important aspect in ecological risk assessment of transgenic oilseed rape. Theoretical studies identified survival in the seed bank as the life history trait with the largest impact on population growth rate and persistence [12,13]. Origin and population dynamics of feral populations of *B. napus *have been studied in Western Europe. Pessel et al. [14] showed that feral oilseed rape persisted on road verges for at least eight years. In southern England most *B. napus *populations were transient and supplied mainly by seed input from trucks in transit [15], whereas in central France the origin of at least 35% of feral populations from the seed bank was estimated by Pivard et al. [11]. In northwest Germany, some feral populations may persist via self-recruitment, as inferred from four years of field survey and assessment of genetic variation [16]. The situation in Central Europe is little known, and population genetics of feral oilseed rape populations have rarely been studied.

To investigate genetic diversity, origin and persistence of feral oilseed rape in Austria, we compared feral populations sampled in various seminatural habitats with 19 commercial varieties which had been widely released in Austria. To assess molecular variation, short sequence repeats (SSR) proved to be best suitable in an initial screening of several molecular marker systems (RAPD, AFLP, SINE, ISSR and SSR) [10]. In the statistical analysis of these data, we complement traditional population genetic methods with the assignment test implemented in the software STRUCTURE [17] in order to obtain information on population structure and gene flow.

## Results

The observed number of alleles (*n*_*a*_) and the observed heterozygosity (*H*_*O*_) were not significantly different among the groups of commercial varieties and feral populations (Table 1, Wilcoxon: *P *> 0.05). The same was true for the effective number of alleles (*n*_*e*_) and Nei's expected heterozygosity (*H*_*E*_) although the values obtained for the feral group were higher than those for the commercial varieties. When all feral individuals were considered in the analysis, a significant difference (Wilcoxon: *P *< 0.05) was found for the normalized Shannon's Index (*I*_nor_) for which the feral group was 47% genetically more diverse than the group of commercial varieties (Table 1). Considering the within-sample variability, only one of the analysed samples (the commercial variety 'Idol') was genetically uniform (*I*_nor _= 0.00, Table 2). In all samples heterozygous individuals were identified although considerable among-sample variation in the observed heterozygosity (*H*_*O*_) was found. When all the 224 individuals were analysed, 85 SSR alleles were detected, of which only 38 (45%) were shared between the commercial varieties and the feral groups (Table 3). A high number of private alleles (i.e. exclusive of each group) was observed both for the commercial varieties (19, 22%) and the feral group (28, 33%). When the 13 single feral oilseed rape plants from different sites were excluded from the analysis, the proportion of shared alleles between the two groups slightly increased (35 out of 74 alleles, 47%) and the number of private alleles of the feral group was reduced from 28 to 17 (20%).

**Table 1:** Locus by locus descriptive genetic statistics, for both commercial varieties and feral groups.

		Commercial Varieties					
**Locus Name**	**Allele ** **Length**	**Sample ** **Size**	***n*_a_**	***n*_e_**	***H*_O_**	***H*_E_**	***I*_nor_**
							
**Na12-C08**	139-219	288	9	1.70	0.13	0.41	0.16
**Na12-E06a**	311-355	286	5	2.54	0.14	0.61	0.20
**Na12-A08(a)**	209-226	292	9	2.09	0.19	0.52	0.20
**Na12-A08(b)**	255-348	294	9	2.78	0.13	0.64	0.23
**Na12-C06**	233-273	294	8	3.06	0.20	0.68	0.24
**Na12-C12**	343-355	284	7	2.20	0.07	0.55	0.21
**Na12-E01(a)**	226-238	276	2	1.78	0.64	0.44	0.11
**Na12-E01(b)**	235-330	162	3	2.00	0.32	0.50	0.15
**Na12-D11**	111-123	294	5	2.57	0.22	0.61	0.19
*Total*			57				
Mean		274	6.3	2.30	0.23	0.55	0.19
*St. dev*.			2.7	0.46	0.17	0.09	0.04
							
		**Feral Populations**					
**Na12-C08**	139-219	128(154)	7(12)	4.15(5.55)	0.09(0.13)	0.76(0.83)	0.34(0.39)
**Na12-E06a**	311-355	128(154)	5 (6)	1.78(2.34)	0.16(0.13)	0.44(0.58)	0.19(0.24)
**Na12-A08(a)**	209-226	128(154)	9(10)	2.86(3.14)	0.16(0.25)	0.66(0.69)	0.29(0.30)
**Na12-A08(b)**	255-348	126(144)	7 (7)	2.82(2.59)	0.17(0.18)	0.65(0.62)	0.27(0.25)
**Na12-C06**	233-273	112(138)	6 (7)	3.85(4.39)	0.16(0.14)	0.75(0.78)	0.33(0.34)
**Na12-C12**	343-355	106(130)	4 (7)	1.99(2.19)	0.00(0.03)	0.50(0.55)	0.19(0.23)
**Na12-E01(a)**	226-238	128(150)	3 (4)	2.11(2.13)	0.86(0.83)	0.53(0.53)	0.17(0.17)
**Na12-E01(b)**	235-330	78 (96)	7 (8)	4.16(4.43)	0.05(0.12)	0.77(0.78)	0.38(0.38)
**Na12-D11**	111-123	126(150)	4 (5)	2.00(1.88)	0.10(0.09)	0.51(0.47)	0.19(0.18)
*Total*			52(66)				
*Mean*		118(141)	5.78(7.33)	2.86(3.18)	0.19(0.21)	0.62(0.64)	0.26(0.28)
*St. dev*.			1.92(2.45)	0.97(1.30)	0.26(0.24)	0.13(0.13)	0.08(0.08)

*n*_*a *_= observed number of alleles; *n*_*e *_= effective number of alleles [51]; *H*_*O *_= observed heterozygosity; *H*_*E *_= Nei's [40] unbiased expected heterozygosity; *I*_nor _= normalized Shannon's Index [41,42]. For feral populations all statistics are given both, considering only the eight populations (64 individuals) or, in brackets, also including the 13 single individuals (77 individuals). For characteristics of the nine *B. napus *SSR-loci used see Lowe et al. [38]. The primer sequences are listed in BrassicaDB http://ukcrop.net.

**Table 2:** Descriptive genetic statistics for commercial varieties samples and feral populations.

Sample name	Sample Size	*n*_a_	*n*_e_	Polymorphic Loci (%)	*H*_O_	*H*_E_	Number of Genotypes	*I*_nor_
**Commercial Varieties**								
'Artus'	8 (15.78)	1.56	1.27	55.6	0.26	0.16	4	0.52
'Cannon'	8 (14.89)	2.00	1.49	66.7	0.31	0.27	7	0.92
'Columbus'	8 (16.00)	1.33	1.16	22.2	0.13	0.10	3	0.35
'Express'	8 (13.78)	1.63	1.26	50.0	0.17	0.17	3	0.35
'Falcon'	8 (15.56)	1.67	1.36	44.4	0.16	0.20	7	0.92
'Fornax'	8 (14.22)	2.38	1.67	87.5	0.25	0.32	8	1.00
'Gazelle'	8 (14.22)	1.25	1.16	25.0	0.13	0.10	2	0.18
'Honk'	8 (15.56)	1.78	1.40	22.2	0.08	0.15	8	1.00
'Idol'	8 (16.00)	1.11	1.11	11.1	0.11	0.06	1	0.00
'Impala'	8 (14.00)	1.63	1.53	62.5	0.24	0.30	8	1.00
'Karola'	8 (16.00)	2.11	1.54	88.9	0.21	0.31	7	0.92
'Lady'	8 (15.78)	1.78	1.25	66.7	0.13	0.18	4	0.52
'Lirajet'	8 (15.56)	1.67	1.51	44.4	0.29	0.24	5	0.67
'Mohican '	8 (14.44)	1.44	1.25	44.4	0.14	0.15	3	0.47
'Panther'	5 (7.78)	2.33	1.82	100.0	0.39	0.55	5	1.00
'Phantom'	6 (9.11)	2.00	1.60	66.6	0.19	0.36	5	0.87
'Pronto'	8 (16.00)	1.56	1.52	55.6	0.43	0.28	4	0.64
'Synergy '	8 (15.78)	2.67	1.65	100.0	0.42	0.39	7	0.35
'Zeus'	8 (14.00)	1.38	1.30	37.5	0.26	0.17	4	0.58
Average	7.74	1.75	1.41	55.33	0.23	0.23	5	0.65
Total	147						90	0.85
								
**Feral Populations**								
# 70	8 (14.67)	2.67	1.83	100.0	0.17	0.47	6	0.80
# 71	8 (15.33)	2.22	1.86	88.9	0.22	0.45	6	0.83
# 74	8 (12.44)	2.22	1.61	62.5	0.17	0.31	6	0.80
# 80	8 (14.67)	2.22	1.79	77.8	0.20	0.38	8	1.00
# 84	8 (15.56)	2.22	1.44	77.8	0.16	0.28	7	0.92
#118	8 (16.00)	2.44	1.64	88.9	0.19	0.35	8	1.00
#128	8 (16.00)	1.22	1.13	22.2	0.13	0.07	2	0.18
#154	8 (13.11)	1.63	1.37	50.0	0.34	0.21	4	0.58
Average	8	2.11	1.58	71.01	0.20	0.32	5.88	0.76
Total	64						47	0.84
								
**13 Feral Genotypes**								
(each of different origin)	13	4.89	2.74	100.0	0.31	0.58	13	1.00
Total	77						60	0.91

*n*_*a *_= observed number of alleles; *n*_*e *_= effective number of alleles [51]; *H*_*O *_= observed heterozygosity; *H*_*E *_= Nei's [40] unbiased expected heterozygosity; *I*_nor _= normalized Shannon's Index [41,42]. Sample size is given both as number of individuals analysed and (between bracket) as average number of data per locus. The incomplete genotyping is due to technical reasons.

**Table 3:** Comparison between commercial varieties and feral populations (64 individuals) of *B. napus *for shared and private SSR alleles.

				Number of Private Alleles	
	Sample Size	Number of Alleles	Number of Shared Alleles	Commercial	Feral
**Na12-C08**	416(442)	13(17)	3 (4)	6 (5)	4 (8)
**Na12-E06a**	414(440)	7 (7)	3 (4)	2 (1)	2 (2)
**Na12-A08(a)**	420(446)	11(12)	7 (7)	2 (2)	2 (3)
**Na12-A08(b)**	420(438)	10(10)	6 (6)	3 (3)	1 (1)
**Na12-C06**	406(432)	9(10)	5 (5)	3 (3)	1 (2)
**Na12-C12**	390(414)	8(10)	3 (4)	4 (3)	1 (3)
**Na12-E01(a)**	404(426)	3 (4)	2 (2)	0 (0)	1 (2)
**Na12-E01(b)**	240(258)	7 (8)	3 (3)	0 (0)	4 (5)
**Na12-D11**	420(444)	6 (7)	3 (3)	2 (2)	1 (2)
Total		74(85)	35(38)	22(19)	17(28)
					
Mean	392(416)	8.22(9.44)	3.89	2.44(2.11)	1.89(3.11)
St. dev.		2.95(3.68)	1.69	1.88(1.62)	1.27(2.15)

For data in parentheses, the 13 sampling sites of single feral individuals are included (altogether 77 individuals).

The difference in allelic frequencies between the group of commercial varieties and the group of the eight feral populations of *B. napus *was moderate and significant (*F*_CT _= 0.132, *P *= 0.0009; Table 4a). When the two groups were considered separately, genetic divergence was strong, both among samples of commercial varieties (*F*_ST _= 0.606, *P *< 0.0001) and among the feral populations (Table 4b, *F*_ST _= 0.466, *P *< 0.0001). Pairwise *F*_ST _values for all combinations of feral populations *versus *samples from commercial varieties ranged from 0.209 to 0.900 (Table 5, with *P *< 0.05 in all cases and *P *< 0.001 in 93% of the cases), i.e. from moderate to nearly complete genetic differentiation, with an average of 0.584 ± 0.143 (strong genetic differentiation). The inbreeding level was lower within varieties (*F*_IS _= -0.020, *P *> 0.05) than within feral populations (*F*_IS _= 0.361, *P *< 0.0001).

**Table 4:** Analysis of molecular variance (AMOVA) for 19 commercial varieties and eight feral populations of *B. napus*.

Source of Variation	***d.f***.	Sum of Squares	Variance Components	Percentage of Variation
**a**				
Among groups	1	86.469	0.360 (V_a_)	13.19
Among samples within groups	25	549.007	1.331 (V_b_)	48.81
Among individuals within samples	184	215.258	0.134 (V_c_)	4.90
Within individuals	211	190.500	0.903 (V_d_)	33.10
Total	421	1041.235	2.727 (V_T_)	
**Fixation Indices**	*F*_IS _= 0.129*	*F*_SC _= 0.562**	*F*_CT _= 0.132**	*F*_IT _= 0.669**
				
**b**				
**Commercial Varieties**				
Among varieties	18	405.929	1.400 (V'_a_)	60.70
Among individuals within varieties	128	113.758	-0.018 (V'_b_)	-0.79
Within individuals	147	136.000	0.925 (V'_c_)	40.09
Total	293	655.687	2.308 (V'_T_)	
**Fixation Indices**	*F*_IS_ = 0.020^*ns*^	*F*_ST _= 0.606**	*F*_IT _= 0.599**	
				
**Feral Populations**				
Among populations	7	143.078	1.164 (V'_a_)	46.64
Among individuals within populations	56	101.500	0.480 (V'_b_)	19.25
Within individuals	64	54.500	0.852 (V'_c_)	34.11
Total	127	299.078	2.496 (V'_T_)	
**Fixation Indices**	*F*_IS _= 0.361**	*F*_ST _= 0.466**	*F*_IT _= 0.659**	

For each group, total molecular variance is partitioned into three components: among groups (commercial varieties *versus *feral populations), among populations within groups and within populations.

*P < 0.001, **P < 0.0001 (based on 100000 permutations).

**Table 5:** Values of *F*_ST _for all possible combinations between feral populations and commercial varieties of *B. napus*.

Feral Populations	Commercial Varieties										
	'**Artus**'	'**Cannon**'	'**Columbus**'	'**Express**'	'**Falcon**'	'**Fornax**'	'**Gazelle**'	'**Honk**'	'**Idol**'	'**Impala**'	
**# 70**	0.615	0.470	0.644	0.595	0.540	0.485	0.648	0.597	0.580	0.477	
**# 71**	0.563	0.469	0.580	0.574	0.440	0.465	0.656	0.595	0.538	0.486	
**# 74**	0.678	0.527	0.720	0.694	0.607	0.578	0.725	0.692	0.660	0.605	
**# 80**	0.636	0.570	0.681	0.671	0.558	0.557	0.680	0.668	0.575	0.577	
**# 84**	0.696	0.585	0.745	0.726	0.610	0.605	0.779	0.701	0.699	0.610	
**#118**	0.673**	0.600	0.690	0.576	0.625	0.534	0.649	0.687	0.558	0.594	
**#128**	0.847	0.681	0.881	0.826	0.835	0.732	0.870	0.824	0.900	0.698	
**#154**	0.747	0.615	0.695	0.661	0.738	0.515	0.689	0.659	0.794	0.541	
**Mean**	0.682	0.565	0.704	0.666	0.619	0.559	0.712	0.678	0.663	0.574	
***s.d***.	0.087	0.073	0.087	0.086	0.121	0.084	0.078	0.072	0.129	0.072	
											
	'**Karola**'	'**Lady**'	'**Lirajet**'	'**Mohican**'	'**Panther**'	'**Phantom**'	'**Pronto**'	'**Synergy**'	'**Zeus**'	**Mean**	***s.d***.
**# 70**	0.396	0.608	0.479	0.575	0.239*	0.310**	0.401	0.353	0.483	0.500	0.118
**# 71**	0.332	0.537	0.464	0.599	0.209*	0.270**	0.373	0.359	0.429	0.470	0.120
**# 74**	0.407	0.667	0.615	0.678	0.355**	0.449	0.532	0.448	0.599	0.591	0.111
**# 80**	0.403	0.660	0.577	0.659	0.218	0.256	0.428	0.350	0.408	0.533	0.146
**# 84**	0.338	0.683	0.603	0.734	0.347	0.439	0.541	0.500	0.580	0.606	0.128
**#118**	0.547	0.680	0.608	0.663	0.315**	0.283**	0.512	0.432	0.496**	0.564	0.117
**#128**	0.694	0.832	0.722	0.837	0.651**	0.715	0.661	0.610	0.794	0.769	0.089
**#154**	0.610	0.681	0.639	0.768	0.503	0.563	0.577	0.480	0.669	0.639	0.092
**Mean**	0.466	0.668	0.588	0.689	0.355	0.411	0.503	0.442	0.557	0.584	0.143
***s.d***.	0.134	0.083	0.084	0.087	0.153	0.164	0.097	0.090	0.130	0.143	

All *F*_ST _values were significant for *P *< 0.001 except where specified: *P < 0.05, **P < 0.01). With the exception of 'Panther' (5) and 'Phantom' (6) sample size was eight for each commercial variety.

**Figure 1:**
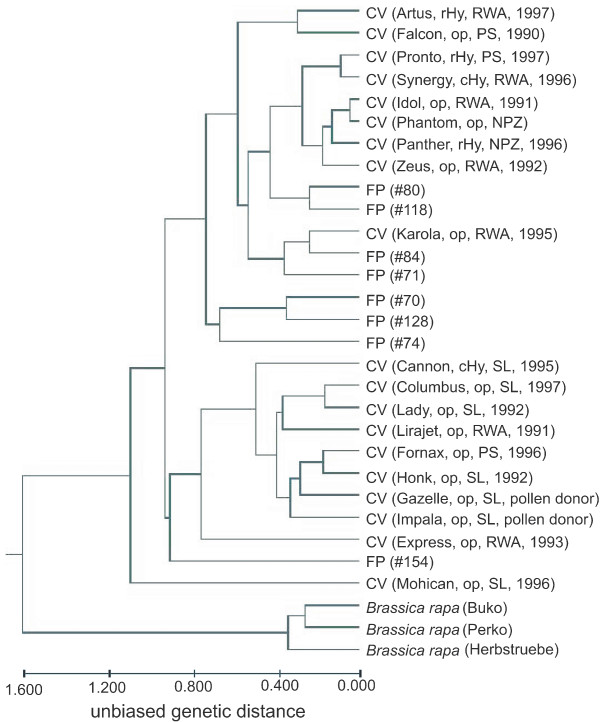
**Relationship among samples based on Nei's **[40]**unbiased genetic distance and UPGMA clustering **[43]. FP = feral populations, CV = commercial varieties; variety type: OP = open pollinated, cHy = composite hybrid, rHy = restored hybrid. Seed company: NPZ = Norddeutsche Pflanzenzucht, PS = Probstdorfer Saatzucht, RWA = Raiffeisen Ware Austria, SL = Saatbau Linz. The year of approval for each variety is also indicated.

In the cluster analysis of samples, *B. rapa *samples were well separated from *B. napus *(Figure 1). Cutting the cluster at a genetic distance of 1.00, two clusters of *B. napus *samples and a singleton could be obtained. The singleton was represented by the variety 'Mohican'. The two clusters correlated partially with variety types and seed-companies. In the lower cluster eight out of nine varieties were open pollinated (OP) (except 'Cannon') and six out of nine were from Saatbau Linz. This cluster also included two varieties ('Gazelle', 'Impala') which served as pollen donors but were never released in Austria. In the uppermost cluster all the restored hybrid cultivar (rHy) varieties were present. Seven of the eight feral populations were included in the upper cluster, with some (#84, #71, #80 and #118) closer to the commercial varieties than others (#70, #128 and #74).

**Figure 2:**
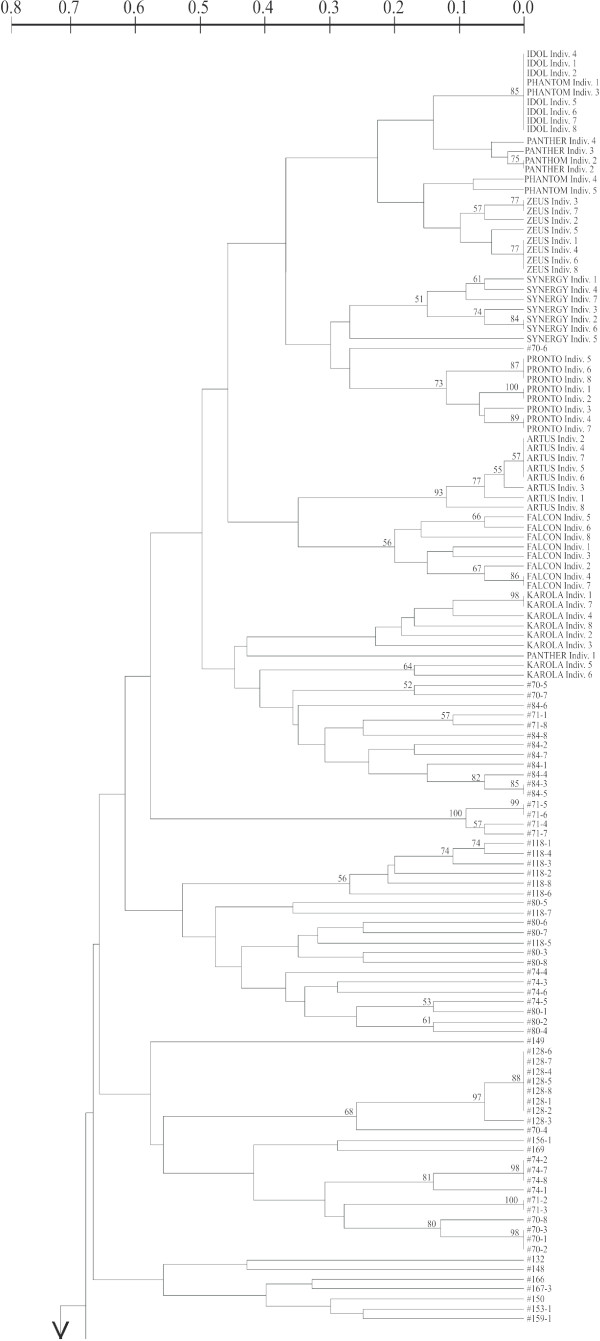
**Relationship among single individuals of *B. napus *based on Nei and Li genetic distances and UPGMA**. For comparison individuals from three commercial varieties of *B. rapa *were also included. Nei and Li [48] genetic distances and UPGMA method implemented in Treecon ver. 1.b software [49] were used. For this analysis each SSR allele was coded as a single locus with two states, 0 (absent) or 1 (present) [50]. The consistency of each node was assayed by bootstrapping (i.e. resampling with replacement) with 1000 replicates using Treecon ver. 1.3b software [49]. Only bootstrap values (%) over 50 are reported in the figure.

**Figure 3:**
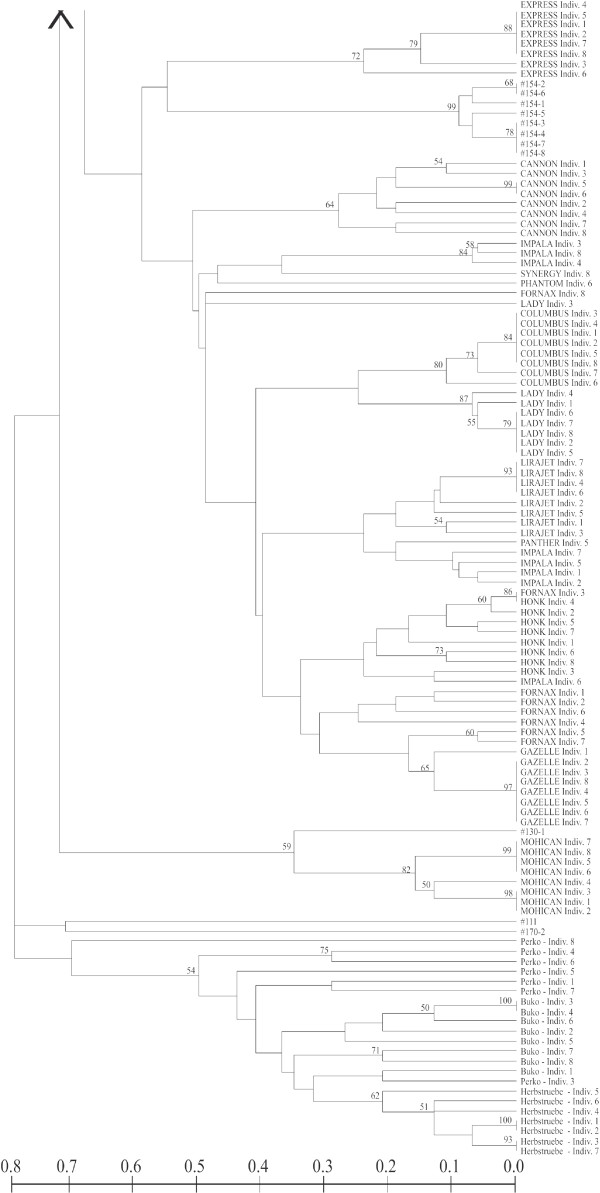
**Relationship among single individuals of *B. napus *based on Nei and Li genetic distances and UPGMA**. Figure 2 continued, for detailed description see Figure 2.

Genotypes of *B. rapa *were also well separated from those of *B. napus *in an UPGMA-tree based on individual genotypes (Figures 2 and 3). Whereas individuals within both commercial varieties and feral populations often shared the same multilocus genotype, no multilocus genotype was shared between commercial varieties and feral populations.

**Figure 4:**
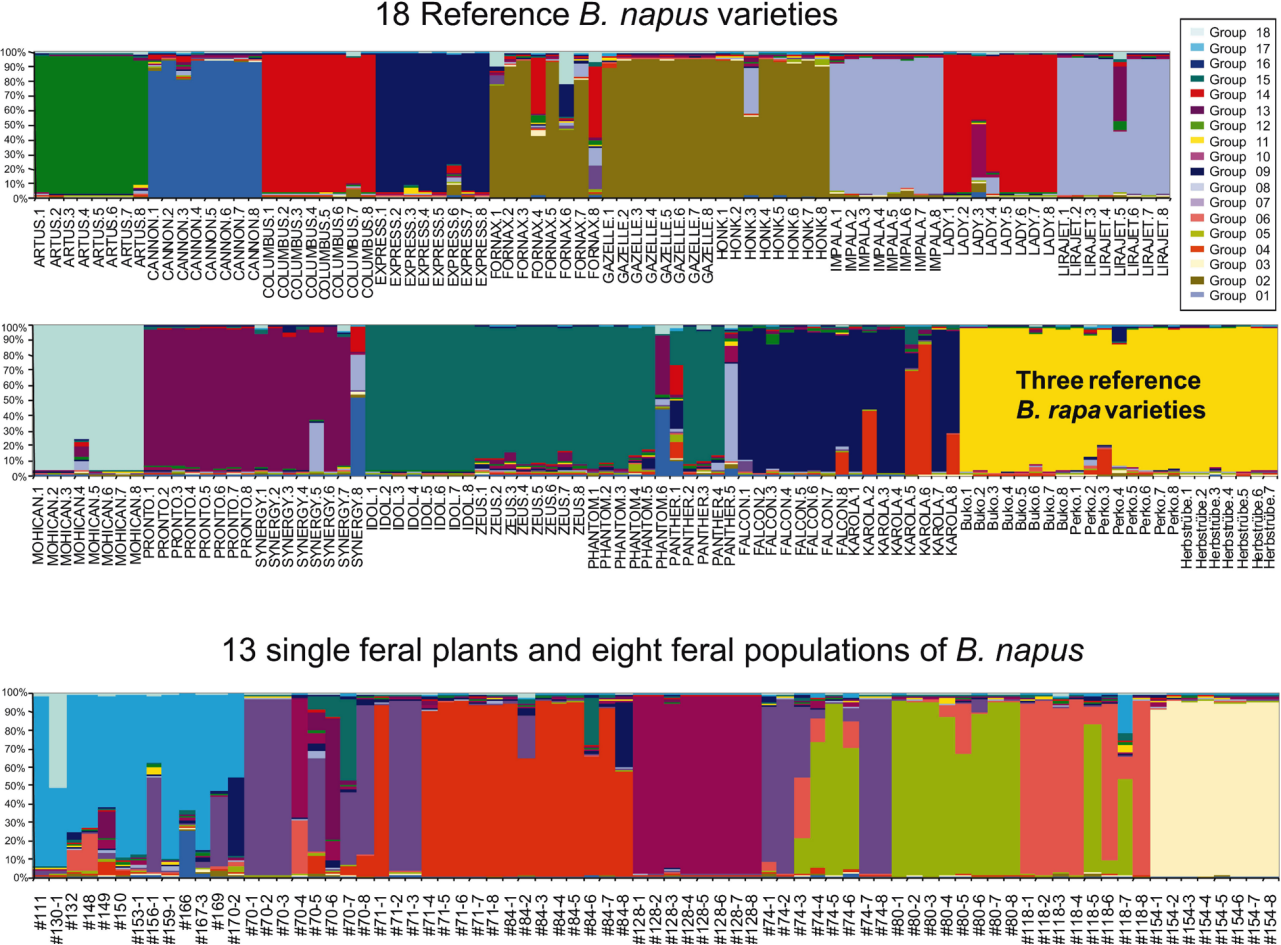
**Results of the cluster analysis using STRUCTURE software**. Different colours represent different genetic groups. Each column represents an individual. The height of the column segments shows the probability of assignment of this individual plant to the genetic groups.

Applying the method of Evanno et al. [18] to identify the most likely number of 'true populations', we found *K *= 18 genetic groups. With one exception -- group 4 was both represented by individuals from a commercial variety ('Karola') and feral populations (#71, #84) -- the assignment procedure of STRUCTURE showed a clear separation between commercial varieties and feral samples (Figure 4). Ten genetic groups were found exclusively among the commercial varieties, each of them comprising one to four varieties. Several variety samples were genetically homogenous, whereas others included some 'deviant' genotypes. Seven genetic groups were resolved among the feral samples, most of them occurring at several sites. Whereas some feral populations appear to be genetically homogeneous (#128, #154) others show a mixture of highly diverse genotypes (notably #70 and #74). Remarkably, the single feral plants, sampled over a wide area, tend to cluster together (7 out of 13 individuals were attributed to the same group with a proportion of membership > 70%) and, despite some individuals were strongly admixed (#156-1,#130-1, #170-2, #169, #149, #166), it is also noticeable that all share some similar genetic characteristics. Both commercial and feral samples include a few individuals which were assigned to two different genetic groups with nearly equal probability, suggestive of a hybrid origin (e.g. individual #130-1, feral group 17 and group 18 otherwise represented by the variety 'Mohican').

## Discussion

The feral populations of *B. napus *appeared to be genetically differentiated from the commercial varieties included in our study. Overall, both groups showed a similar population genetic make-up with a similar level of genetic diversity both within and between samples. Commercial oilseed rape varieties and feral populations, however, shared less than 50% of the SSR alleles and not a single multilocus genotype. A moderate level of genetic differentiation, 13% of the total molecular variation, was observed between the two groups of plants. None of the eight feral populations could be related to any investigated variety, although some showed similarities to certain cultivars: most plants from samples #71 und #84 cluster with the variety 'Karola', which appears genetically heterogeneous itself.

Microsatellite (SSR) markers are suitable to identify varieties of oilseed rape [19]. We cannot exclude that small sample sizes and seed contamination (as inferred by the observed heterogeneity of several variety samples) may have limited our ability to detect genetic contributions of some varieties to the feral populations. In seed production, varietal purity of oilseed rape can only be achieved with great effort due to pollination from uncontrolled sources [20] [K. Fischer, personal communication]. In Austria, the purity of oilseed rape varieties is not controlled on a genetic basis but only by checking morphological characteristics [21].

Founding events and genetic drift could have a significant role in shaping the population genetic structure of feral populations. The rather large genetic differences among feral populations and the comparatively high level of inbreeding of the feral individuals support this hypothesis. Some feral populations appeared to be genetically fairly homogeneous, suggesting their origin from a single founding event. Other sites show highly heterogeneous genetic composition, suggesting that they received seed input from several sources, and probably over several years. The genetically most diverse stand of feral oilseed rape was found at a railway embankment (#70), other mixed populations were sampled along roads (#74, #118) and a river bank (#71), all habitats likely to receive repeated influx of seeds and to experience repeated disturbance.

Several genetic clusters of feral plants occurred at sites separated by large distances, the single plants sampled from mostly small feral populations were assigned to the same cluster. These genetic similarities among feral plants could be caused by common ancestry from varieties that are no longer cultivated, by selection favouring or eliminating certain alleles of loci linked to the markers, or perhaps also by hybridization with related species. In any case, the data suggest that these feral populations already existed for several years. Thus, our findings corroborate the results obtained by Pessel et al. [14], using chemical compounds and isozyme analyses, of at least eight years persistence of feral *B. napus *in France. Pessel et al. [14] mainly observed homogeneous sites with all plants showing the same genotype, but 23% of their sites contained a mixture of plants with different genotypes and/or hybrid genotypes. In our study, all feral sites contained more than one genotype. This difference can be explained by the high resolving power of SSR markers in comparison with the chemical compounds and isozyme analyses or by more frequent outcrossing events in our study region. Recently, Elling et al. [16] observed high diversity of multilocus genotypes (using a subset of the SSR loci scored in our study) in feral *B. napus *populations in northwest Germany; 58% percent of the feral plants could be assigned to one of seven reference varieties. The detection of hybrids between varieties and of many private alleles in feral plants indicates persistence of feral populations, an outcome that mirrors our results.

Several lines of evidence suggest that containment of transgenic oilseed rape will be hard to achieve. Colonization success of feral populations of *B. napus *critically depends on human or natural disturbances [10,15,22]. Volunteers of oilseed rape in arable fields are common, and have been found by Austrian plant breeders for at least twelve years after the last oilseed rape crop [H. Schrems, personal communication]. Volunteers of genetically modified herbicide resistant oilseed rape have already been observed up to ten years after field trials [23,24]. Long range seed dispersal could be caused by road traffic [25,26]. In Canada, where herbicide resistant varieties have been cultivated at large scale, volunteers as well as feral populations with multiple herbicide resistance occur, whose origin is attributed to pollen flow among cultivars with different resistance traits [5,27]. Introgression of a herbicide resistance transgene into a weedy population of *B. rapa *has already occurred in Canada [28]. Hybridization of *B. napus *has also been observed with its other parent species, *B. oleracea *[29], and with several other related species [8,30].

Small scale agriculture typical for Austria is characterized by a variety of landscape elements separating small fields [31]. In these agrotopes, numerous feral oilseed rape plants can be found as well as many related species of Brassicaceae, both weedy crops and native species [22]. The abundance and flowering phase of these species differ among regions even in a small country such as Austria [10]. Therefore, detailed taxonomic and ecological knowledge of the regional flora is vital for risk assessment of genetically modified plants [32]. Even when hybridization between crops and their wild relatives is rare, introgression of transgenes can have pervasive effects on the wild populations, especially if they confer a fitness advantage to the plants carrying them, as can be expected from traits contributing to pest resistance or stress tolerance [9,33].

At present, coexistence of farming with genetically modified (GM) and non-GM crops is vehemently debated [22,34,35]. In this context, feral populations of crops play an important role as sources and corridors for gene transfer [14]. In view of the persistence of feral populations, as suggested by our results, and all the available evidence on seed survival, gene flow and introgressive hybridization of oilseed rape, coexistence of GM and non-GM oilseed rape can hardly be achieved without compromising the interests of GM-free agriculture. These problems for coexistence are particularly important in Austria with its small scale landscape structure and a high proportion of organic farmers opposed to using transgenic crops.

## Conclusions

The genetic divergence between feral samples and commercial varieties indicates that feral oilseed rape is able to maintain persistent populations. These feral populations have to be considered in ecological risk assessment and coexistence measures as a relevant hybridisation partner of transgenic oilseed rape.

## Methods

### Sampling of plant material and DNA extraction

**Figure 5:**
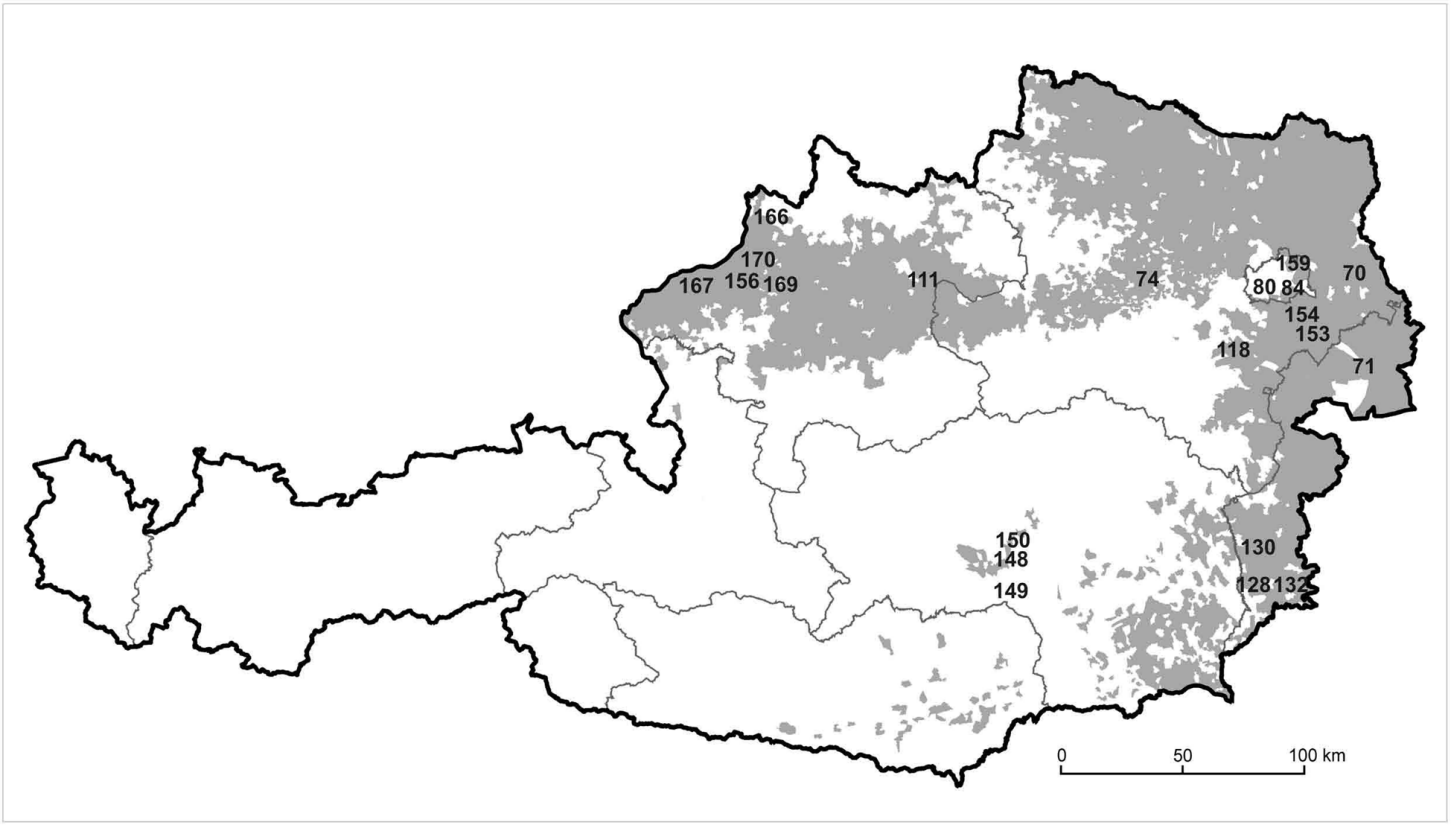
**Locations of the sampling sites on the map of Austria**. The locations of the sampling sites of the eight analysed feral populations and of the thirteen single plants (sampling numbers) are shown. The grey shading indicates the cultivation areas of oilseed rape, lines show the borders of the provinces of Austria.

Following a survey of feral oilseed rape populations in Austria [10], eight feral *B. napus *populations consisting of more than ten plants were selected for genetical investigation, chosen to maximize habitat diversity (Table 6; Figure 5). We sampled eight individuals per population. In addition, thirteen single oilseed rape plants -- each from a different sampling site -- were also included for a total of 77 feral individuals to be genetically analysed. Fresh and cold treated leaves (4°C) were used for DNA-analyses. Fieldwork was carried out during spring, summer and autumn in 1998 and 1999. Seeds from 19 oilseed rape commercial varieties, as well as three varieties of *B. rapa*, which were widely grown in Austria from 1990 to 1999 were obtained from Austrian and German breeders and seed trading companies (Table 7). Seeds of commercial varieties were germinated and the apex of the secondary leaves of the young plants was used for DNA extraction with Cetyltrimethyl-Ammonium-Bromid (CTAB) method [36]. The extraction protocol followed Doyle and Doyle [37].

**Table 6:** Characterization of the investigated plant material (feral oilseed rape; OSR) and its sampling sites.

Analysed Plants	Sampling Number	Number of Individuals Analysed	Estimated Population Size	Sampling Location	Characterization of Sampling Sites
***B. napus***	# 70	1-8	15	Lower Austria, Lassee	railway embankment
**Feral**	# 71	1-8	25	Burgenland, Podersdorf	along a wet river bank
**Populations**	# 74	1-8	30	Lower Austria, St. Pölten	verge of a motorway
	# 80	1-8	20	City of Vienna	excavated soil in front of a new building
	# 84	1-8	25	Vienna, Danube Island	excavated soil
	#118	1-8	30	Lower Austria, Pfaffstätten	traffic island
	#128	1-8	several 100s	Burgenland, Rohr im Burgenland	approximately five year old fallow
	#154	1-8	30	Lower Austria, Hexenbühel	loess embankment
***B. napus***	#111	1	small	Upper Austria, Enns	road verge in front of a petrol station
**Single** **Feral Plants**	#130	1	30	Burgenland, Rohr im Burgenland	maize field (volunteer)
	#132	1	single plant	Burgenland, Rohr im Burgenland	field margin, 10 m next to an OSR field
	#148	1	big	Styria, near Großlobming	bean field (volunteer)
	#149	1	single plant	Styria, Großlobming	road verge
	#150	1	very big	Styria, Großlobming	ripe OSR field, second blossoming
	#153	1	<50	Lower Austria, Hexenbühel	fallow
	#156	1	5	Upper Austria	road embankment
	#159	1	15	Vienna, Danube Island	sidewalk verge
	#166	1	several	Upper Austria, near Schärding	strawberry field
	#167	1	5	Upper Austria, near Stuben	road verge adjacent to a maize field
	#169	1	several	Upper Austria, near Eggerding	field (volunteer)
	#170	1	5	Upper Austria, near Forchtenau	road verge

**Table 7:** Oilseed rape and *Brassica rapa *commercial varieties widely grown in Austria from 1990 to 1999.

Name of Commercial Variety	Pollination System	Breeders and Seed Trading Companies	Date of Approval
***Brassica napus***			
'Artus'	rHy	RWA	05.08.1997
'Cannon'	VA 75 hybrid, cHy	SL	04.08.1995
'Columbus'	o. p.	SL	05.08.1997
'Express'	o. p.	RWA	16.12.1993
'Falcon'	o. p.	PS	18.12.1990
'Fornax'	o. p.	PS	06.08.1996
'Gazelle'	o. p.	SL	pollen donor
'Honk'	o. p.	SL	17.12.1992
'Idol'	o. p.	RWA	17.12.1991
'Impala'	o. p.	SL	pollen donor
'Karola'	o. p.	RWA	19.12.1995
'Lady'	o. p.	SL	17.12.1992
'Lirajet'	o. p.	RWA	17.12.1991
'Mohican'	o. p.	SL	06.08.1996
'Panther'	rHy	NPZ	1996
'Phantom'	o. p.	NPZ	-
'Pronto'	rHy	PS	05.08.1997
'Synergy'	cHy	RWA	19.12.1996
'Zeus'	o. p.	RWA	1992
			
***Brassica rapa***			
'Buko'	o. p.	PS	17.12.1981
'Perko PVH'	o. p.	ÖRWZ	15.12.1967

'Herbstrübe'	o. p.	undefined variety	undefined variety

(source: Österreichische Sortenliste 1998)

rHy: restored hybrid, cHy: composite hybrid, o. p.: open pollinated;

RWA: Raiffeisen Ware Austria, SL: Saatbau Linz, PS: Probstdorfer Saatzucht, NPZ: Norddeutsche Pflanzenzucht, ÖRWZ: Österreichische Raiffeisen Warenzentrale.

### SSR-analyses (Simple Sequence Repeats)

Thirty-nine SSR primers were tested [38]. Nine loci which were polymorphic and with stable amplification were used in the present study (Table 3). Scoring of genotypes was conducted via PCR with fluorescent primers [39] followed by DNA-fragment separation with an automatic DNA-sequencer on the basis of capillary electrophoresis (ABI 373 and ABI 310 DNA sequencer from Applied Biosystems).

### Statistical analyses

To evaluate the level of genetic diversity of both commercial varieties and feral *B. napus *populations the following statistics were calculated: the percentage of polymorphic loci, the number of alleles (*n*_*a*_, per locus and average), the effective number of alleles (*n*_*e*_, per locus and average), the expected (*H*_*E*_, [40]) and the observed (*H*_*O*_) heterozygosity, the number of unique genotypes and the normalized Shannon's Index (*I*_nor_, either with allele frequencies at a locus or the frequencies of multilocus genotypes [41,42]). The softwares PopGene ver. 1.31 [43] and TFPGA ver 1.3 [44] were used. The significance of the differences between the groups of commercial varieties and that of feral populations were tested using the Wilcoxon non parametric test implemented in JMP ver 3.1.5 software [45].

In order to evaluate population structure, the total genetic variation (V_T_) was partitioned performing the Analysis of Molecular Variance (AMOVA, [46]) with hierarchical levels. The analysis was repeated considering the two groups (commercial variety and feral populations) separately. The pairwise *F*_ST _matrix among samples was also obtained and the significance of each *F*_ST _was calculated with 3.000 permutations. All calculations were made by using Arlequin ver. 2.0 software [47].

Relationships among samples were studied by obtaining a dendrogram applying UPGMA method based on a matrix of Nei [40] pairwise genetic distances using PopGene ver. 1.31 [43]. Twenty-seven samples of *B. napus *(19 of commercial varieties and eight of feral populations) and three samples of *B. rapa *were included in this analysis. Relationships among the individuals were described with a dendrogram by UPGMA method based on Nei and Li [48] pairwise genetic distances matrix.

Population structure was also inferred using the Bayesian method implemented in STRUCTURE Ver. 2.1 software [17], that infers the number of clusters (populations), *K*, present in a sample by comparing the posterior probability for different numbers of putative populations specified by the user and assigns individuals giving a percentage of membership (q) to these clusters. Twenty independent runs for each *K *(from 1 to 21) were performed using 100,000 MCMC repetitions and 100,000 burn-in periods, using no prior information and assuming correlated allele frequencies and admixture. The number of clusters (*K*) was estimated computing the *ad hoc *statistic Δ*K*, based on the rate of change in the log probability of the data between successive *K *values [18].

## Authors' contributions

KP, JG and GGr designed and organized this research, KP and HR obtained the samples, SM carried out the molecular assays, DR performed the statistical analyses and contributed to the writing of the paper. DR, GGo and KP provided interpretation of results, KP and GGo wrote the paper. All authors read and approved the paper.
